# Genomic insights into Wnt signaling in an early diverging metazoan, the ctenophore *Mnemiopsis leidyi*

**DOI:** 10.1186/2041-9139-1-10

**Published:** 2010-10-04

**Authors:** Kevin Pang, Joseph F Ryan, James C Mullikin, Andreas D Baxevanis, Mark Q Martindale

**Affiliations:** 1 Kewalo Marine Laboratory, Pacific Biosciences Research Center, University of Hawaii at Manoa, Honolulu, HI, USA; 2 Genome Technology Branch, National Human Genome Research Institute, National Institutes of Health, Bethesda, MD, USA; 3 NIH Intramural Sequencing Center, National Human Genome Research Institute, National Institutes of Health, Bethesda, MD, USA

## Abstract

**Background:**

Intercellular signaling pathways are a fundamental component of the integrating cellular behavior required for the evolution of multicellularity. The genomes of three of the four early branching animal phyla (Cnidaria, Placozoa and Porifera) have been surveyed for key components, but not the fourth (Ctenophora). Genomic data from ctenophores could be particularly relevant, as ctenophores have been proposed to be one of the earliest branching metazoan phyla.

**Results:**

A preliminary assembly of the lobate ctenophore *Mnemiopsis leidyi *genome generated using next-generation sequencing technologies were searched for components of a developmentally important signaling pathway, the Wnt/β-catenin pathway. Molecular phylogenetic analysis shows four distinct Wnt ligands (*MlWnt6*, *MlWnt9*, *MlWntA *and *MlWntX*), and most, but not all components of the receptor and intracellular signaling pathway were detected. *In situ *hybridization of the four Wnt ligands showed that they are expressed in discrete regions associated with the aboral pole, tentacle apparati and apical organ.

**Conclusions:**

Ctenophores show a minimal (but not obviously simple) complement of Wnt signaling components. Furthermore, it is difficult to compare the *Mnemiopsis *Wnt expression patterns with those of other metazoans. mRNA expression of Wnt pathway components appears later in development than expected, and zygotic gene expression does not appear to play a role in early axis specification. Notably absent in the *Mnemiopsis *genome are most major secreted antagonists, which suggests that complex regulation of this secreted signaling pathway probably evolved later in animal evolution.

## Background

**Figure 1:**
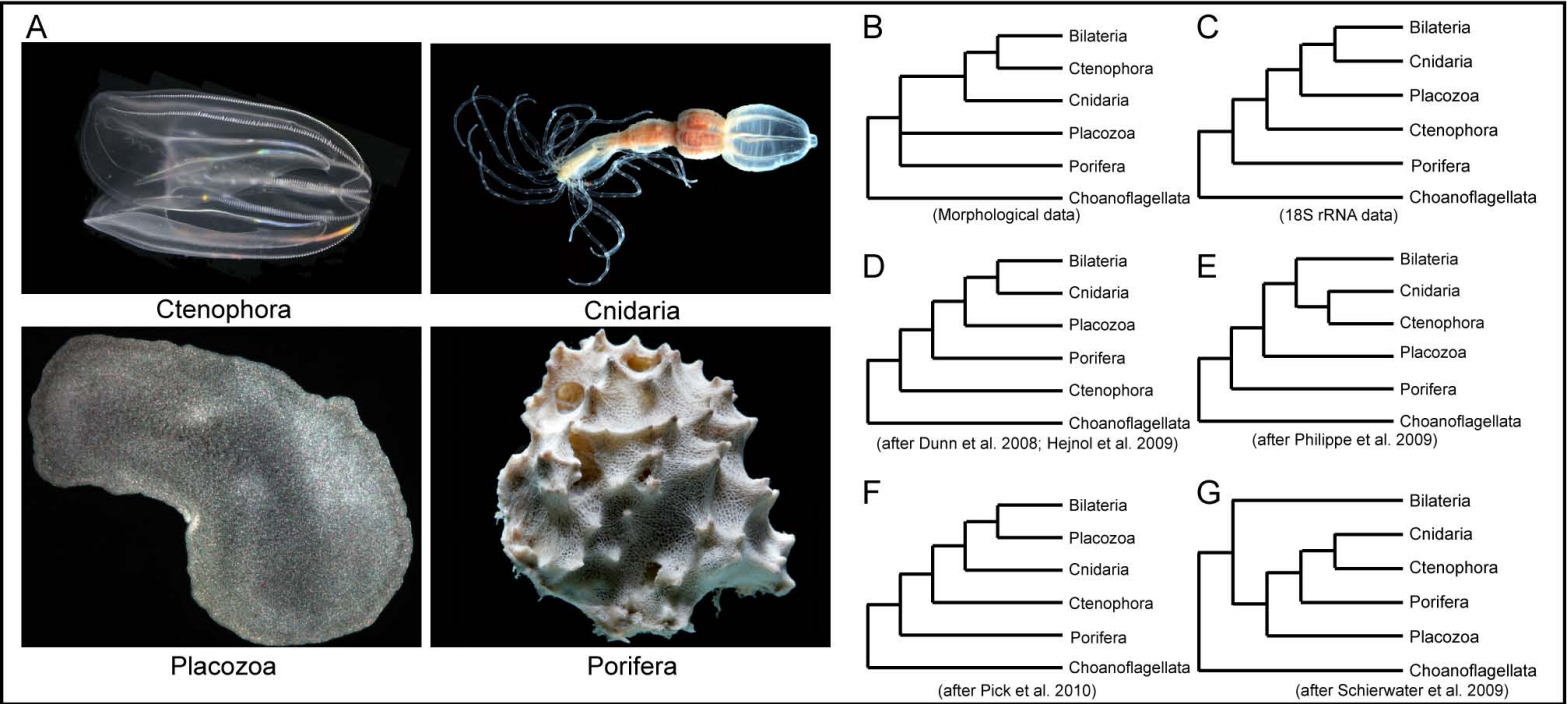
**Non-bilaterian animal relationships**. **(A) **Representative images of non-bilaterian animals, Ctenophora (*Mnemiopsis leidyi*), Cnidaria (*Nematostella vectensis*), Placozoa (*Trichoplax adhaerens*) and Porifera (*Dysidea *spp.). Photos courtesy of William E. Browne and Eric Roettinger. **(B-G) **Alternate hypotheses on early animal evolution and the placement of the ctenophores, based on **(B) **morphological data, **(C) **18S ribosomal RNA results, **(D-F) **different phylogenomic analyses and **(G) **a combined morphological and phylogenomic approach.

The overwhelming majority of phylogenetic studies identify four clades of metazoan animals that branched off before the origin of the Bilateria. These include cnidarians (corals, sea anemones and 'jellyfish'), poriferans (sponges), placozoans (*Trichoplax*) and ctenophores (comb jellies) (Figure 1A). Often referred to as 'basal metazoans', 'diploblasts' or non-bilaterians, these four clades display radically different adult body plans and developmental programs from one another. The exact relationship of these early-branching taxa to one another remains contentious. Although morphological data suggest that poriferans and placozoans were the earliest metazoan lineages, followed by cnidarians and then ctenophores (Figure 1B) [1,2], molecular studies have led to a number of different hypotheses regarding early animal evolution. Studies using 18S ribosomal RNA have suggested that sponges were the earliest branch, followed by ctenophores, thereby making them more basal compared with the classification based on morphological studies (Figure 1C) [3-6]. With the dawn of phylogenomics, the position of the ctenophores has continued to be contentious. The ctenophores have been positioned as sister to all other metazoans (Figure 1D) [7,8], grouped with the cnidarians in a clade known as the Coelenterata (Figure 1E) [9], and considered sister to the clade comprising Bilateria, Placozoa and Cnidaria (Figure 1F) [10]. Additionally, a combined morphological and phylogenomic analysis has even suggested a monophyly of the basal metazoans in the clade 'Diploblastica', which is sister to the Bilateria (Figure 1G) [11]. As yet, there is very little consensus as to the placement of Ctenophora in the animal tree of life.

Fortunately, genomic data (gene content and complexity) and information on overall genomic structure can prove useful in resolving the relationship of these clades to one another. The genomes of the anthozoan cnidarian *Nematostella vectenis *[12], the hydrozoan cnidarian *Hydra magnipapillata *[13], the placozoan *Trichoplax adhaerens *[14] and the sponge *Amphimedon queenslandica *[15] have already proven to be invaluable resources in the effort to understand the genomic makeup of the earliest metazoans. Along with data from other sponges [16], the genomic data from choanoflagellates [17,18] (the sister group of metazoans) have provided significant insight into the molecular complexity present in the closest extant unicellular ancestor of animals. Nonetheless, the available data from ctenophores (that is, the modest expressed sequence tag (EST) sets from two species, *Mnemiopsis leidyi *and *Pleurobrachia pileus*) is far from sufficient to resolve the placement of this enigmatic lineage.

**Figure 2:**
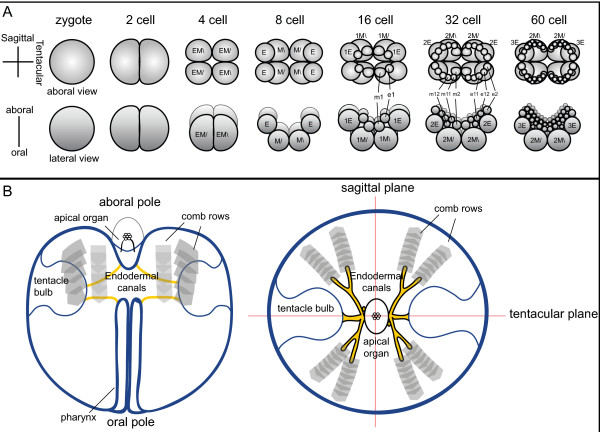
**Ctenophore development and body plan**. **(A) **Early cleavage from egg to 60-cell stage, based on Martindale and Henry [20] and others. The top row shows the view from the aboral (or vegetal) pole and the bottom row shows the lateral view, with the oral pole at the bottom. The first two divisions are equal and meridional, and the third cleavage is unequal and oblique, giving rise to middle (M) and end (E) macromeres. Subsequent divisions are unequal, with micromeres given off at the aboral pole. **(B) **Basic ctenophore body plan, as shown during the cydippid stage. The oral pole is the location of the mouth, which opens to the pharynx. The pharynx leads internally to the gut and endodermal canals (yellow). Also shown are paired tentacle bulbs (from which the tentacles grow), the eight comb rows, and the apical sensory organ located at the aboral pole.

Unlike the other non-bilaterians, ctenophores display a stereotypical developmental program (Figure 2A), with a well-studied cell lineage [19,20]. The first two cleavages are equal and meridional, whereas the third cleavage is unequal and oblique. At this stage, the eight macromeres divide unequally to give off micromeres at the future aboral pole. Many of the early blastomeres in ctenophore embryos display a precocious determination of cell fate that is consistent with segregation of cytoplasmic determinants, although some inductive interactions are known to occur [21]. Unfortunately, no good molecular candidates for cell fate specification determinants have been identified in ctenophores. The primary adult body axis, the oral--aboral axis, is established at the time of the first cleavage [22] and early cleavages are important for localizing developmental potential [23]. The oral-aboral axis of larval (or cydippid) and adult ctenophores is demarcated by the mouth at the oral pole and the apical sensory organ at the aboral pole (Figure 2B). Additionally, there are two planes of rotational symmetry: the tentacular plane, which passes through the two tentacles, and the oesophageal or sagittal plane, which is perpendicular to the tentacular plane. Ctenophores also possess complex features, such as a well-developed muscular system composed of non-epithelial muscle cells and a nervous system that comprises sensory cells and a subepidermal nerve net [reviewed in [24]].

Although ctenophores have proven to be exceptional experimental embryological material, very little is known about the identity of the exact genes and proteins involved in specifying the body axes. To date, work on ctenophores has focused mainly on different families of transcription factors, including Sox [25], Fox [26], T-box [27] and Homeobox [28,29], yet nothing is known about the cell signaling pathways. Bilaterian model systems have identified a limited number of cell signaling pathways, including the Wnt/β-catenin, TGF-β, Hedgehog, Notch, receptor tyrosine kinase, and Jak/STAT pathways. These pathways generally involve an extracellular (and often diffusible) ligand, transmembrane receptor, intracellular signal transduction/amplification system and, interestingly, a system of antagonists that can be used to further regulate informational content. These systems are used repeatedly in different tissues throughout the life history of organisms [30], with the basic elements of these systems arising early in animal evolution [31].

**Figure 3:**
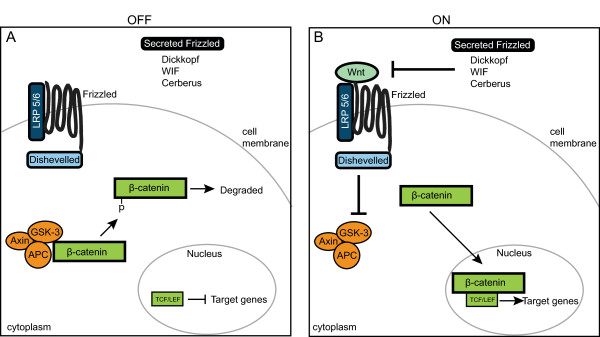
**Overview of Wnt/β-catenin signaling Ctenophore pathway**. **(A) **When Wnt signaling is inactive, cytoplasmic β-catenin protein is bound by the 'destruction complex' of axin, glycogen synthase kinase 3 (GSK-3) and adenomatous polyposis coli (APC). While sequestered, GSK-3 phosphorylates β-catenin, which targets β-catenin for ubiquitination and degradation. **(B) **In the presence of a Wnt ligand, the pathway is activated. Wnt binds to the seven-transmembrane receptor Frizzled and its co-receptor lipoprotein receptor-related protein 5/6 (LRP5/6), which causes Dishevelled (Dsh) to be activated. Dsh inhibits GSK-3, thereby allowing β-catenin to accumulate in the cytoplasm. Eventually, β-catenin gets translocated to the nucleus, where it interacts with the transcription factor T-cell-specific transcription factor/lymphoid enhancer binding factor (TCF/LEF) to activate target genes. The diffusible antagonists (Secreted Frizzled-related (Sfrp), Dickkopf (DKK), Wnt Inhibitory Factor (WIF) and Cerberus (CER)) can modulate Wnt activity by preventing the binding of Wnt to its receptors.

In this study we examined the Wnt/β-catenin signaling pathway in the ctenophore *Mnemiopsis leidyi *(Figure 3). In this pathway, the absence of a Wnt ligand results in the shunting of cytoplasmic β-catenin into a 'destruction complex' of axin, adenomatous polyposis coli (APC) and glycogen synthase kinase 3 (GSK-3) [32]. GSK-3 phosphorylates specific residues in the amino terminus of β-catenin, thereby targeting β-catenin for degradation via ubiquitination. T-cell-specific transcription factor/lymphoid enhancer binding factor (TCF/LEF) interacts with the repressor Groucho to suppress specific target genes. When the Wnt ligand is present, it activates the signaling cascade by first binding to the seven-transmembrane receptor Frizzled (Fzd). Along with a co-receptor, lipoprotein receptor-related protein 5/6 (LRP5/6), Wnt binding results in the phosphorylation of Dishevelled (Dsh), thereby activating it. Dsh inhibits GSK-3 activity, which allows active, non-phosphorylated β-catenin to accumulate in the cytoplasm. Increasing levels of cytoplasmic β-catenin promotes translocation to the nucleus, where it interacts with TCF/LEF (and other cofactors) to enhance transcription of target genes. Recent work in a number of cnidarian species has shown that the Wnt pathway is evolutionarily highly conserved and plays important roles in axis and cell fate specification [33-40]. Work in the sponge *Amphimedon *has shown polar localization of a Wnt ligand, suggesting a role in axial specification [41]. In another species of sponge, *Oscarella lobularis*, Wnt/β-catenin signaling has been implicated in adult epithelial patterning and ostia formation [42]. Some components of this pathway are known to be present in *Trichoplax *[14,31], but their expression patterns and function are not yet known.

We recently used next-generation technologies to sequence the genome of the lobate ctenophore *Mnemiopsis leidyi*, in an effort to better understand early animal evolution. In this paper, we look at one particular aspect, the evolution of the canonical Wnt signaling pathway. We found a near-complete Wnt signaling pathway present, including four Wnt ligands. However, part of the 'destruction complex' appears to be incomplete, and many Wnt antagonists are not recognizable in the genome. *In situ *hybridization studies showed that transcripts for all four Wnt genes are detected relatively late in development in discrete domains of the developing tentacles and apical organ.

## Results

### Wnt/β-catenin pathway

A preliminary assembly of the genome of the lobate ctenophore *Mnemiopsis leidyi*, totalling 156 megabases in 5,100 scaffolds was generated by the National Institutes of Health (NIH) Intramural Sequencing Center using 454 and Illumina sequencing techniques. Using reciprocal BLAST searches of the *Mnemiopsis *genome, reverse transcriptase PCR cloning, and subsequent phylogenetic analyses, we identified and isolated nearly all of the essential members of the canonical Wnt/β-catenin signaling pathway (Table 1).

**Table 1:** Wnt/β-catenin pathway members present in the *Mnemiopsis leidyi *genome

**Gene name**^**a**^	Mle scaffold or accession number	Pfam domains	**E value**^**b**^	**Human hit**^**b**^
WntA	HM448813	Wnt	7e^-46^	NP_110388.2: Wnt4

Wnt6	HM448814	Wnt	5e^-48^	NP_006513.1: Wnt6

Wnt9	HM448815	Wnt	7e^-59^	CAA96283.1: Wnt13

WntX	HM448816	Wnt	4e^-56^	NP_110388.2: Wnt4

β-catenin	HM448817	ARM(12)	2e^-109^	BAG70078.1: Beta-catenin

Dishevelled (Dsh)	HM448818	DIX, PDZ, DEP	8e^-67^	AAB65244.1: DSH3

FrizzledA	HM448819	Fz, Fri 7TM	2e^-89^	NP_003498.1: Frizzled7

FrizzledB	HM448820	Fz, Fri 7TM	5e^-34^	NP_003498.1: Frizzled7

Sfrp^c^	HM448821	Fz	9e^-20^	AAD41636.1: Frizzled1

TCF^d^	HM448822	HMG	2e^-51^	CAB97214: TCF4

Pygopus	HM448823	PHD zinc finger	3e^-17^	CAB43209.1: Pygo2

Chibby	HM448824		3e^-6^	CAQ07904.1: Chibby1

Porcupine	HM448825	Transmembrane	2e^-24^	NP_073736.2: Porc isoA

DIXD^e^	HM448826	DIX	9e^-17^	AAH41626.2: DIXDC1

LRP5/6^f^	**c600800006.Contig20**	EGF(2), LY, EGF, LY(4), EGF	5e^-27^	BA3305.1: LRP5

GSK-3^g^	**c606000111.Contig2**	Ser/Thr kinase	4e^-156^	NP_001139628.1: GSK-3 beta

APC^h^	c408602200.Contig1	ARM(2)	2e^-17^	AAF01784.1: APC2

CK1^i^	**c608300381.Contig1**	Ser/Thr kinase	3e^-127^	EAW61775.1: CK1
	
	**c607500101.Contig1**	Ser/Thr kinase	9e^-106^	CAA56710.1: CK1

Tankyrase	**c606800096.Contig3**	ANK(10), SAM, PARP, C2	0.0	NP_003738.2: Tankyrase-1

CBP^j^	**c601400002.Contig7**	ZnF, KIX, BROMO, DUF906, ZnF	0.0	AAC51770.1: CREB-binding protein

Groucho/TLE^k^	**c601900005.Contig5b20**	WD40(6), LDLa(3), transmembrane	4e^-108^	AAH43247.1: TLE3

Wntless/evi	**c607300052.Contig1**	transmembrane	1e^-11^	BAC11072.1: wntless

Lzic/Icat	**c606200080.Contig2**		1e^-20^	NP_115774: LZIC

^a^Provisional gene names and scaffolds of *Mnemiopsis *Wnt pathway members, along with GenBank Accession numbers of those cloned.

^b^Also listed is the best human BLAST hit with corresponding E value.

^c^Sfrp = Secreted Frizzled-related;

^d^TCF = T-cell-specific transcription factor

^e^DIXD = DIX domain containing protein

^f^LRP = lipoprotein receptor-related protein

^g^GSK-3 = glycogen synthase kinase 3

^h^APC = adenomatous polyposis coli

^i^CK1 = casein kinase 1

^j^CBP = CREB binding protein

^k^TLE = transducin-like enhancer of split

**Figure 4:**
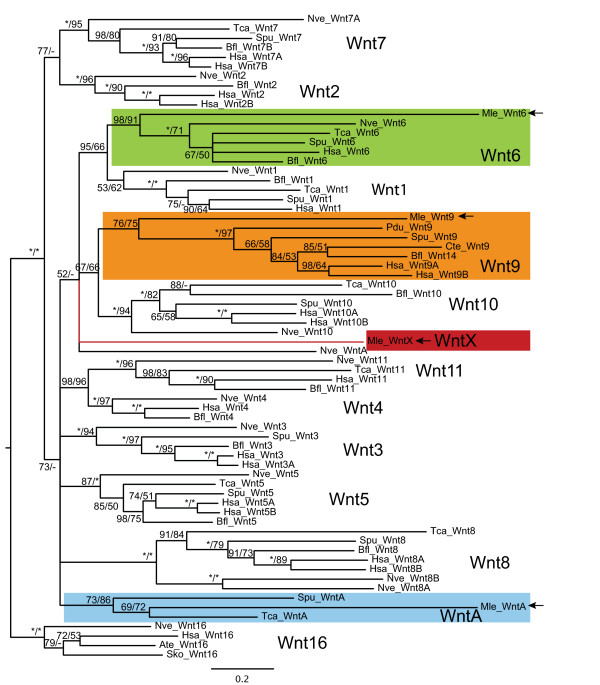
**Wnt gene orthology**. Bayesian analysis to determine orthology of *Mnemiopsis *Wnt genes. Shown is a consensus tree of four independent runs of 5 million generations each. Posterior probability support is shown at each node, as well as maximum likelihood (PhyML with WAG model) bootstrap support (posterior probability/ML bootstrap). Asterisks at each node indicate ≥ 99%. support. A dash (-) indicates that the node was not supported by maximum likelihood analyses. *Mnemiopsis *Wnt genes are shown shaded and marked with arrows. Ate = *Archaearanea tepidariorum*; Bfl = *Branchiostoma floridae*; Cte = *Capitella teleta*; Hsa = *Homo sapiens*; Mle = *Mnemiopsis leidyi*; Nve = *Nematostella vectensis*; Pdu = *Platynereis dumerilii*; Sko = *Saccoglossus kowalevskii*; Spu = *Strongylocentrotus purpuratus*; Tca = *Tribolium castaneum*.

The four non-paralogous Wnt genes identified in the *Mnemiopsis *genome were cloned by rapid amplification of cDNA ends (RACE) PCR, using mixed-stage embryonic cDNA. Phylogenetic analyses including the sequences of Wnt genes from representative taxa having fully annotated genomes showed moderately strong support for the ctenophore Wnt genes grouping in the *Wnt6*, *Wnt9 *and *WntA *families (Figure 4A). The fourth Wnt gene *MlWntX *does not group with any of the recognized Wnt families. It should be noted that the ctenophore Wnt genes tend to be somewhat more divergent than other animal genomes, as evidenced by their longer branch lengths in our phylogenies, and this is consistent with previous analyses of homeobox genes ([29] and Ryan *et al*, submitted). In addition, except for *MlWnt6*, the support for the other Wnt genes is relatively low. When phylogenetic analyses included additional non-bilaterian taxa (such as *Amphimedon*, *Oscarella *and *Trichoplax*), family-level classification of some *Mnemiopsis *sequences showed variation, perhaps due to artifacts caused by long branch attraction (see Additional file 1). The genomic complement of Wnt genes varies greatly across the Metazoa; however, there are 13 well-supported and described families, with a few orphans [40,43]. The four *Mnemiopsis *Wnt genes are comparable in number with *Amphimedon *and *Trichoplax*, which both possess three, whereas cnidarians and bilaterians possess nearly the full complement of Wnt genes [35,43].

**Figure 5:**
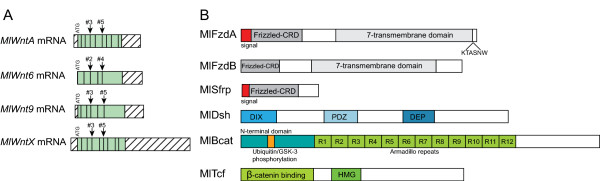
**Wnt gene structure and domains of Wnt pathway members**. **(A) **Intron-exon structure of the four Mnemiopsis Wnt transcripts that were cloned. Turquoise shading indicates the coding region and the diagonal lines show the 5' and 3' untranslated regions. The start (ATG) is indicated, and the vertical lines represent intron positions. The conserved intron positions are marked with arrows. **(B) **Predicted protein domains present in the other Wnt components that were cloned out. Specific domains and other regions of interest are colored and named.

Each *Mnemiopsis *Wnt gene has a predicted signal peptide at the 5' end. *MlWntA*, *MlWnt9 *and *MlWntX *have the 24 conserved cysteine residues, whereas *MlWnt6 *has only 22. Comparison of the coding regions with that of genomic data reveals two important intron positions (Figure 5A) that are well conserved across all metazoan Wnt genes analyzed to date [43]. These correspond to introns 3 and 5 in *MlWntA*, *MlWnt6 *and *MlWnt9*, and to introns 2 and 4 in *MlWntX*. In the current assembly, none of the Wnt genes are on the same scaffold, so there is no evidence of genomic linkage. *MlWntA *is currently on a 182 kb scaffold, spanning positions 64,343 to 70,583, with predicted genes 4 kb upstream (highest BLAST hit was XP_001624925.1) and 10 kb downstream (XP_002018102). *MlWnt9 *is on a 283 kb scaffold, spanning 131,863 to 139,362, with adjacent genes located 4 kb upstream (XP_001620894.1) and 12 kb downstream (XP_002755989.1). *MlWntX *is on a 285 kb scaffold, region 26,134 to 12,946, with no genes detected downstream and the closest hit 12 kb upstream (ZP_03967055). *MlWnt6 *is currently on a 48 kb contig with no other genes predicted, spanning the region 35,863 to 8,886. Extrapolating, the closest possible distance between any two genes is 20 kb.

### Receptors and downstream components of the Wnt signaling pathway

In addition to the four Wnt ligands, the *Mnemiopsis *genome contains the receptors Fzd and LRP5/6 (Table 1). We were able to clone and identify two Fzd genes (*MlFzdA *and *MlFzdB*), both containing the extracellular Frizzled cysteine-rich domain (CRD), which binds Wnt ligands, and the transmembrane domain (Figure 5B). *MlFzdA *also has a signal peptide and the intracellular KTXXXW motif (KTASNW), which is thought to bind the PDZ domain of Dsh and is therefore required for canonical Wnt signaling [44]. *MlFzdB *does not appear to have the signal peptide or the KTXXXW motif based on cloned RACE PCR fragments. There was only a single LRP5/6 identified in the genome.

For functional canonical Wnt signaling, key intracellular components of the pathway are required. In *Mnemiopsis*, there are single genes encoding *Dishevelled *(*MlDsh*) and *β-catenin *(*MlBcat*). MlDsh contains all three key domains found in Dishevelled proteins of other animals (DIX, PDZ and DEP) (Figure 5B). The full-length *MlBcat *sequence was cloned from a mixed stage cDNA template. Similar to β-catenin from other metazoans, there is a highly conserved GSK-3 phosphorylation site and a conserved N-terminal motif (Figure 5B). Centrally, there are 12 armadillo repeats that are clearly detectable but widely divergent compared with other metazoan sequences. Surprisingly, based on the homology of predicted protein sequences, MlBcat appears to lack both C-terminal motifs (motifs A and B), which are thought to serve as transactivational domains [45]. When Wnt signaling is inactive, the 'destruction complex', composed of axin, APC and GSK-3, binds cytoplasmic β-catenin and targets it for degradation [32]. Although we found a clear GSK-3 ortholog, *in silico *searches found only a partial match to APC (low similarity to armadillo repeat domain and lacking all other domains) and did not find any evidence of axin. It is known that GSK-3 can phosphorylate β-catenin without requiring the other members of the complex [46]. We did find that the transcription factor TCF/LEF (*MlTcf*), the binding partner of stabilized nuclear β-catenin, is required for the activation of downstream target genes. MlTcf contains the β-catenin binding domain at its amino terminus and also contains the the Sox-Tcf high mobility group domain, which binds DNA (Figure 5B).

Although we found Wnt pathway genes from all parts of the pathway, including ligand modification/secretion, receptors and other membrane-associated proteins, and cytoplasmic and nuclear factors (Table 1; see Additional file 2), we failed to identify the important antagonists Dickkopf (DKK), Wnt Inhibitory Factor (WIF) and Cerberus (CER), which are characteristic of bilaterian Wnt signaling. We were able to identify a possible Secreted Frizzled-related gene (*MlSfrp*) that may be involved in regulating Wnt signaling; it contains the extracellular Frizzled CRD but not the transmembrane domain (Figure 5B). Unlike bilaterian Sfrp, MlSfrp lacks a Netrin-like (NTR) domain.

### Wnt/β-catenin expression patterns

**Figure 6:**
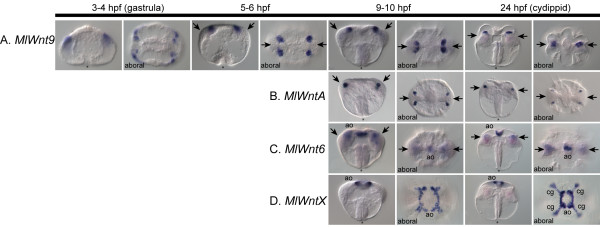
**Wnt gene expression during development**. Whole- mount *in situ *hybridization analyses of all Wnt genes during development. The timeline at the top depicts hours post-fertilization (HPF). All images are oriented laterally, unless otherwise specified (aboral). For lateral views, the oral pole is at the bottom and aboral pole at the top, with an asterisk (*) marking the blastopore/mouth. For the aboral views, the tentacular plane is horizontal and the sagittal plane vertical. Gene expression is detected colorimetrically and shown by the blue/purple staining. **(A) ***MlWnt9 *is first detected after gastrulation in four aboral regions of the future tentacle bulb (arrows). After approximately 9 HPF, these four groups of cells converge along the tentacular plane and form two groups of cells within the tentacle bulb. **(B) ***MlWntA *is also expressed in four groups of cells of the forming tentacle bulb, slightly more internal than *MlWnt9*. It remains expressed in these four groups of cells at the periphery of the tentacle bulb. **(C) ***MlWnt6 *is expressed in both the tentacle bulb and the floor of the apical organ (ao). The tentacular staining is fainter in the cydippid stage; however, the apical organ staining remains prominent. **(D) ***MlWntX *is expressed both in the apical organ floor and in the ciliated groove (cg), which is the structure that connects the apical organ to the individual comb rows.

We examined the mRNA expression patterns of key components of the Wnt signaling pathway by whole-mount *in situ *hybridization. All four Wnt genes are detected at relatively late stages of development after gastrulation. *MlWnt9 *is detected the earliest, at about 3 to 4 hours post-fertilization (HPF) in four rows of cells in the aboral ectoderm derived from micromeres born at the vegetal pole (Figure 6A). As development proceeds, these cells form four clusters within the forming tentacle bulb, which appear to approach the aboral midline and fuse into two groups. Similarly, *MlWntA *is expressed in four groups of cells in the anlage of the tentacle apparati, beginning at about 9 HPF (Figure 6B). These cells are located below the surface ectoderm and are adjacent to the forming tentacle bulb, in the most lateral regions. In comparison with the *MlWnt9*+ cells, these are located slightly deeper below the surface and are positioned more towards the distal extremes of the tentacular axis.

Whereas *MlWntA *and *MlWnt9 *are primarily expressed in small regions of the tentacle bulb, the other two Wnt genes are associated with the apical sensory organ and its surrounding regions. The apical organ is primarily a gravity-sensing organ [47], although it possibly also acts as a photoreceptor [48], and it is highly innervated, as evidenced by ultrastructural analysis [24]. *MlWnt6 *is expressed in the apical organ floor, primarily in a central region along the tentacular plane (Figure 6C). There is also faint diffuse expression of *MlWnt6 *within the tentacle bulb. *MlWntX *is expressed in a region surrounding the floor of the apical organ, except for two areas in the pharyngeal plane, where it is excluded (Figure 6D). There is also expression in cells of the ciliated groove, the ciliated connection pathway between the gravity-sensing cells of the apical organ, and the locomotory comb rows.

**Figure 7:**
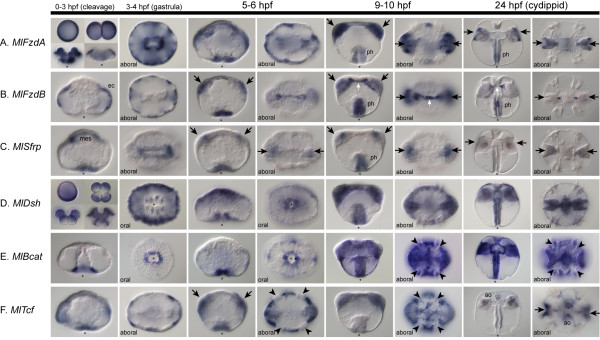
**Expression of Wnt pathway components**. Whole-mount *in situ *hybridization of other members of the Wnt pathway, including **(A) ***MlFzdA*, **(B) ***MlFzdB*, **(C) ***MlSfrp*, **(D) ***MlDsh*, **(E) ***MlBcat *and **(F) ***MlTcf*. The timeline above the images denotes the different stages of embryos below, from 0-3 hours post-fertilization (if applicable) to 24 HPF or the cydippid stage. Unless noted, all images are lateral views, with the asterisk marking the blastopore or mouth. Blue/purple staining shows where the genes are expressed. **(A) ***MlFzdA *is detected uniformly from egg, through early cleavage stages and gastrulation. From 9 HPF onward, it is expressed mainly in the tentacle bulb (arrows) and pharynx (ph). **(B) ***MlFzdB *is not detected until 3-4 HPF in cells of the ectoderm (ec). At 5-6 HPF, it is expressed in the tentacle bulb and around the blastopore, in cells that will invaginate to form the pharynx. Later, it is additionally expressed in the trans-tentacular muscle (white arrow), which connects the two tentacles. **(C) ***MlSfrp *is expressed in the invaginating pharynx and in the presumptive mesoderm (mes). This mesodermal expression becomes confined to two regions of the tentacle bulb, which becomes barely detectable in the cydippids. The pharyngeal expression is also not detected in cydippid stages. **(D) ***MlDsh *is expressed uniformly from egg to cydippid stages. **(E) ***MlBcat *is first detected after gastrulation (about 4 HPF) in ectodermal cells around the blastopore. This blastoporal expression continues however, at 6 HPF there is *MlBcat *expression everywhere, except in the cells that form the comb plates (arrowheads). **(F) ***MlTcf *is expressed primarily in the ectoderm after gastrulation but excluded from cells that form the comb plates. At cydippid stages, it is expressed in discrete regions of the apical organ floor (ao) and in the tentacle bulbs.

To understand to which cells these Wnt ligands are signaling, we also looked at the expression patterns of the Frizzled-related genes and other intracellular components (Figure 7). *MlFzdA *is expressed maternally, in cleavage stages, and through gastrulation in a uniform manner (Figure 7A). After gastrulation and through cydippid formation, expression becomes concentrated primarily in the pharynx, tentacle bulb, and two ectodermal domains between the comb rows in the sagittal plane. *MlFzdB*, which lacks the intracellular motif, is initially expressed after gastrulation in the ectoderm (Figure 7B). However, later in development, most of the ectodermal expression is downregulated (except in the pharynx), and there is an additional expression domain in the muscle cells that connects the two tentacle apparati. The Secreted Frizzled-related gene, *MlSfrp*, is expressed after gastrulation in the pharynx and also in the mesoderm, which becomes two diffuse regions of the tentacle bulb (Figure 7C). By the cydippid stage, only faint tentacle bulb staining can be detected.

The key Wnt modulator *MlDsh *is expressed maternally in a uniform pattern and throughout development in almost all cells (Figure 7D). By contrast, *MlBcat *is first detected during mid-gastrulation, and is localized to the region surrounding the blastopore (Figure 7E). However, the blastopore, which corresponds to the animal pole and the future location of the mouth, is already fully formed, and the endodermal macromeres have already been internalized by the time zygotic transcripts are detectable. The cells that express *MlBcat *remain on the surface, and expression is not seen in endodermal precursors. We are not able to detect any transcripts before this stage by *in situ *hybridization; however, we cannot rule out that there are low levels of expression or maternally deposited protein present. As development proceeds, *MlBcat *is expressed almost ubiquitously in both the ectoderm and the endoderm. The only region in which it is not expressed (or is expressed at low levels) is in the cells that form the comb plates. It is possible that this widespread expression is due to the role of β-catenin in cell adhesion. The onset of *MlBcat *expression occurs earlier than that of all four Wnt genes and, in contrast to the expression of *MlBcat*, which is initially localized to the oral (animal) pole, all four Wnt genes are localized primarily to the aboral (vegetal) pole of the embryo. Finally, the transcription factor *MlTcf *is expressed after gastrulation diffusely in the ectoderm and more intensely around the blastopore (Figure 7F). Similar to *MlBcat*, it also is not expressed in cells that form the comb plates. Late expression of *MlTcf *is confined to individual cells of the apical organ and parts of the tentacle bulb.

## Discussion

To date, existing studies have offered only a partial view of a limited number of gene families in ctenophores [25-29,49-51]. Using next-generation sequencing, we were able to investigate complete gene families and signaling pathways in the ctenophore *Mnemiopsis leidyi*. Although results from full genome analyses of gene families are not yet available, we examine in this paper the comprehensiveness of an important developmental signaling pathway: the Wnt/β-catenin pathway. To ensure that the genomic searches were complete and that false negatives were minimized, we examined the published 15,752 *Mnemiopsis *ESTs and found that there was a match of approximately 97 to 99% to genomic contigs, depending on stringency conditions (data not shown), suggesting that our genomic sequencing is fairly complete.

### Ancestral metazoan gene complement

When compared with other animals (Table 2), *Mnemiopsis *appears to have a *Wnt *complement that is more similar to poriferans (3 genes) and *Trichoplax *(3) than to cnidarians and bilaterians (7-20), which have up to 13 distinct Wnt family members. This suggests that the expansion of Wnt genes occurred after the divergence of ctenophores, poriferans and *Trichoplax*, and that this expansion was nearly complete in the cnidarian-bilaterian ancestor. However, if *Trichoplax *is the sister group of bilaterians [10], this would suggest a significant loss of Wnt genes in the placozoan lineage. The uncertainty of assigning orthology of sponge Wnt genes [42] and to some of the ctenophore genes makes it difficult to unequivocally determine the evolutionary history of the Wnt gene family. Wnt genes have yet to be found in any non-metazoan (including choanoflagellates), indicating that these ligands are likely to be specific to metazoans. There is not enough phylogenetic information to ascertain the branching order within the Wnt family; however, there is moderate support for the groupings of Wnt1 and 6, Wnt2 and 7, Wnt4 and 11, and Wnt9 and 10, based on our own analyses (Figure 4) and on those performed by others [40,43], suggesting these families resulted from early duplications.

**Table 2:** Wnt/β-catenin pathway evolution

	**Eukaryote**^**a**^	*Mnemiopsis*	*Amphimedon*	*Trichoplax*	*Nematostella*	*Drosophila*	*Homo*
Wnt (total)^b^	-^c^	+ (4)	+ (3)	+ (3)	+ (14)	+ (7)	+ (20)

Frizzled	+^d^	+	+	+	+	+	+

LRP5/6^e^	+	+	+	+	+	+	+

Dishevelled	-	+	+	+	+	+	+

β-catenin	*^f^	+	+	+	+	+	+

Axin	-	-	+	+	+	+	+

APC^g^	-	*	*	+	+	+	+

GSK-3^h^	+	+	+	+	+	+	+

CK1^i^	+	+	+	+	+	+	+

TCF/LEF^jk^	-	+	+	+	+	+	+

Groucho	-	+	+	+	+	+	+

Sfrp^l^	-	+	+	-	+	+	+

DKK^m^	-	-	+^n^	-	+	-	+

WIF^o^	-	-	-	-	-	+	+

CER^p^	-	-	-	-	-	-	+

^a^Refers to non-metazoan eukaryotes. Frizzled (including EAL71080), β-catenin (AAG17931), CK1 (AAD01192) and GSK-3 (P51136) are present in *Dictyostelium*. LRP5 is reported from *Aspergillus *(CAK47340). Other taxa include genome scans of *Mnemiopsis leidyi *(Ctenophora), *Amphimedon queenslandica *(Porifera), *Trichoplax adhaerens *(Placozoa), *Nematostella vectensis *(Cnidaria) and the bilaterians *Drosophila melanogaster *and *Homo sapiens*.

^b^Numbers represent total number of Wnt genes, other rows present presence, absence or partial matches.

^c^Plus sign indicates absence.

^d^Minus sign indicates presence

^e^LRP = lipoprotein receptor-related protein

^f^Asterisk represents partial match.

^g^APC = adenomatous polyposis coli

^h^GSK-3 = glycogen synthase kinase 3

^i^CK1 = casein kinase 1

^j^TCF = T-cell-specific transcription factor

^k^LEF = lymphoid enhancer binding factor

^l^Sfrp = Secreted Frizzled-related

^m^DKK = Dickkopf

^n^DKK has been reported in *Oscarella carmela *(EB741373) but not in *Amphimedon queenslandica*.

^o^WIF = Wnt inhibitory factor

^p^CER = Cerberus

Core components of the Wnt pathway are present in all non-bilaterians (Table 2) [31], suggesting the existence of functionally active signaling at the base of the animal tree of life. Many genes in the Wnt pathway appear to be animal-specific novelties. However, proteins containing Armadillo repeats (such as those in β-catenin) are found in all eukaryotes; these proteins have cytoskeletal functions in fungi and protists, and are involved in intracellular signaling in plants [52]. Certain pathway components are also present in organisms such as the slime mold *Dictyostelium*, which contains a *β-catenin*-like gene called *aardvark*, a GSK-3 homolog and Frizzled-like receptors [53,54]. Aardvark has 10 armadillo repeats (compared with 12 in β-catenin), potential N-terminal GSK-3 phosphorylation sites, and no C-terminus motifs. Functional work has shown *aardvark *to have roles in both adherens junctions and cell signaling (in the form of stalk formation); however this is independent of GSK-3 activity [55]. All other metazoan β-catenin proteins examined to date have the two C-terminus motifs (A and B) that are thought to be transactivational domains, except for *Caenorhabtidis elegans*, which seems to have lost or modified them [45]. Thus, depending on the phylogenetic positions, ctenophores either lost these two motifs or they evolved after the ctenophore divergence. Additionally, bilaterian β-catenins also have other C-terminal motifs, which appear to be lineage-specific innovations.

The lack of certain Wnt pathway components in *Mnemiopsis *that are present in other non-bilaterians is an intriguing result. For instance, axin is found in *Amphimedon*, *Trichoplax*, *Nematostella and *bilaterians, but appears to be missing from *Mnemiopsis *(Table 2). Whether this gene appeared after ctenophores diverged from later metazoan lineages or was lost in the *Mnemiopsis *lineage is not yet clear. Likewise, there seems to be a paucity of diffusible antagonists in *Mnemiopsis*, *Amphimedon *and *Trichoplax*. Whereas *Amphimedon *has several Sfrp-like genes [15,31], *Mnemiopsis *has only a single Sfrp; however, in both species the netrin domain is lacking. A DKK ortholog has been reported only for the sponge *Oscarella carmela *[18], as well as cnidarians and deuterostomes [56]. *Trichoplax *does not appear to have any of the known antagonists. Whereas DKK appears to be relatively ancient and lost in the protostome lineage, WIF is probably a bilaterian novelty and CER is only found in vertebrates. It is likely that antagonists were relatively recent additions to the pathway, providing an extra mechanism to control the activity. Alternatively, there could be additional novel antagonists in *Mnemiopsis *or in the other early lineages, whose identities can only be discovered through functional experiments.

Based on the gene content observed in the early-branching phyla, we can begin to deduce the key steps that led to the complexity observed in the bilaterian Wnt signaling pathway. It appears that the core components were present in the metazoan ancestor, including a Wnt ligand, Frizzled receptor, Dsh and β-catenin. Before the cnidarian-bilaterian ancestor developed, a series of duplication and divergent events, especially among the Wnt genes themselves, led to significant expansion of the components in the pathway. This expansion, coupled with the origin of the Wnt antagonists DKK, WIF and CER was probably the catalyst for the acquisition of additional roles of the pathway.

Based on gene content and diversity, our results are incongruent with a sister relationship between cnidarians and ctenophores (that is, the Coelenterata hypothesis). Firstly, in our phylogenies the genes of ctenophores do not group closely with those of cnidarians. Moreover, if cnidarians and ctenophores were sister phyla, a tremendous amount of gene loss (including the loss of multiple Wnt ligands, axin and DKK) would have been required in the *Mnemiopsis *lineage. These results are consistent with previous analyses of homeobox and nuclear receptor gene families (Ryan *et al*., submitted; Reitzel *et al*., submitted) in rejecting the Coelenterata hypothesis. Unfortunately, the comparison of Wnt signaling components between *Mnemiopsis*, *Amphimedon *and *Trichoplax *is not sufficient to identify the relationships between Ctenophora, Porifera and Placozoa. Additionally, it is not known how well *Mnemiopsis *represents the ancestral ctenophore gene complement, therefore data from other ctenophores would be of great benefit.

### Expression patterns

In *Mnemiopsis*, all of the Wnt genes are expressed at the aboral (vegetal) pole in a striking pattern that suggests they are playing some role in patterning the body. However, they are expressed at such a late stage in development that many cell fates have already been specified. The expression patterns of the Wnt genes in the apical organ and tentacle bulb would suggest they might be involved in neural specification. In cnidarians, it has been suggested that Wnt genes expressed in staggered ectodermal and endodermal domains are patterning the oral-aboral axis in a 'Wnt code' [35,56]. By contrast, most of the Wnt genes in *Nematostella *are primarily expressed at the oral pole, whereas in *Mnemiopsis*, they are expressed at the aboral pole. In the sponge *Amphimedon*, a Wnt gene is expressed at the posterior pole of the swimming larvae [41]. A major similarity is that Wnt genes appear to be expressed at the posterior pole of most animals [57,58]. *Mnemiopsis *locomotes primarily with the oral end to the front (as do most ctenophores), as this aids in feeding; however, they are capable of moving in both directions. Cnidarians, such as *Nematostella*, swim in the direction of the aboral end, as this is the location of their apical tuft. The observed Wnt expression patterns could suggest that the aboral pole of ctenophores corresponds to the posterior pole of bilaterians.

It is difficult to determine whether the Wnt/β-catenin pathway is functioning early in development based on *in situ *expression patterns alone. Whereas *MlFzdA *and *MlDsh *are both expressed maternally and persist through early cleavage, *MlBcat *and the *Wnt *genes are not expressed until after gastrulation. Furthermore, whereas the *Wnts *are detected primarily at the aboral pole, *MlBcat *is initially expressed at the oral (or animal) pole. Either this pathway is not involved in early axis specification or there may be a maternal β-catenin protein that is functioning before onset of zygotic expression. Protein localization (particularly that of β-catenin) would help to determine whether the pathway is involved in axial patterning; however, we have not generated an antibody to β-catenin and have yet to find one that crossreacts. Because MlBcat appears to lack transactivational domains (at least as determined by sequence comparison), further experiments are necessary to determine whether it can actually function as a transcriptional activator. Attempts to activate Wnt signaling via GSK-3 inhibition (for example, with lithium chloride or alsterpaullone treatments) have not produced any obvious phenotype [Pang K, personal observation]. Functional experiments to knock down gene expression (morpholino antisense oligonucleotides or dominant negative constructs) would provide much-needed insight into whether canonical Wnt signaling is actually active in the developing embryo.

## Conclusions

The canonical Wnt signaling pathway evolved at the base of the animal tree of life. We searched through the genome of the ctenophore *Mnemiopsis leidyi*, and identified most of the components of this well-known developmental signaling pathway. Conspicuously absent from ctenophores is axin, a member of the 'destruction complex', which is present in all other animals. Wnt antagonists also appear to be lacking or scarce in early diverging metazoans, with Sfrp present only in ctenophores, sponges and cnidarians, and DKK present only in sponges and cnidarians, with vertebrates possessing the entire array of Wnt antagonists (Sfrp, DKK, WIF and CER). Wnt genes evolved early in animal evolution, but did not radiate and diversify until the Cnidarian-Bilaterian ancestor. However, it is also not clear if Wnt signaling has direct effects on the regulation of gene expression in ctenophores, as key transactivational domains in a downstream target of the Wnt pathway, β-catenin, appear to be absent, and pharmacological treatments that lead to the stimulation of β-catenin activity in other metazoans produce no visible phenotype with these.

Although most of the canonical Wnt pathway components are present, their mRNA expression patterns would suggest that this pathway is not involved in early axis specification in *Mnemiopsis*. Both the late expression patterns (after the axes have been specified) and the expression of *Wnt *and *β-catenin *at opposite poles of the embryo suggest that this pathway may not required for fate specification. The rapid development of ctenophores could imply that asymmetric segregation of maternally loaded protein, rather than zygotic gene expression, is responsible for precocious cell fate specification in these embryos. Further genomic, expression and functional analyses are necessary to determine what genes and/or determinants are involved in axis specification in this unique early diverging animal lineage. Moreover, once the *Mnemiopsis *axial patterning system has been deciphered, it will become increasingly important to reach a consensus regarding the branching position of Ctenophora relative to other early-branching metazoans to place this unique developmental program within a phylogenetic context.

## Materials and methods

### Animal collection and gene expression

*Mnemiopsis leidyi *adults were collected (from Eel Pond or the NOAA Rock Jetty, Woods Hole, MA, USA) during the months of June and July and spawned as previously described [59]. RNA was extracted from embryos at regular intervals from fertilization to 36 hours (TRI Reagent; Molecular Research Center, Cincinnati, OH, USA) [59]. RNA was reverse transcribed to generate cDNA (SMART RACE cDNA Amplification Kit; BD Biosciences, San Jose, CA, USA). This cDNA was used as template to isolate the genes of interest. The following genes were isolated and fully sequenced, and are described in this paper: *MlWntA *(HM448813), *MlWnt6 *(HM448814), *MlWnt9 *(HM448815), *MlWntX *(HM448816), *MlBcat *(HM448817), *MlDsh *(HM448818), *MlFzdA *(HM448819), *MlFzdB *(HM448820), *MlSfrp *(HM448821) and *MlTcf *(HM448822). Additionally sequences were isolated for *MlPygopus *(HM448823), *MlChibby *(HM448824), *MlPorc *(HM448825) and *MlDIXD *(HM448826).

For whole-mount *in situ *hybridization, embryos were fixed at various stages from freshly collected uncleaved embryos (0 HPF) to cydippids (24 to 36 HPF). They were stored in methanol at -20°C until used. Digoxygenin-labeled riboprobes (0.1 ng/ul) (Ambion/Applied Biosystems, Austin, TX, USA) were hybridized for 48 hours at 60°C, and detected using an alkaline phosphatase-conjugated antibody (Roche Applied Science, Indianapolis, IN, USA) and the colorimetric substrate nitro-blue tetrazolium (NBT)/5-bromo-4-chloro-3-indolyl-phosphate (BCIP) [59]. After detection, specimens were washed with phosphate-buffered saline and transferred through a glycerol series up to 70% glycerol. They were then mounted, viewed under a compound microscope (Zeiss Axioskop 2, Jena, Germany), and imaged using a digital imaging system (AxioCam HRc with Axiovision software; Zeiss). Color balance and brightness were adjusted using Photoshop software (Adobe Systems Incorporated, San Jose, CA, USA). The only modification to the *in situ *protocol was a change in acetic anhydride treatment (treated in 0.1 mol/L triethanolamine rather than 1% w/v) (for most recently updated protocols, contact the authors). All *in situ *images presented here and additional developmental stages and/or views, are available online via the comparative gene expression database, Kahikai http://www.kahikai.com.

### Genome sequencing and searches

*Mnemiopsis *genomic DNA was collected from the self-fertilized spawning of two separate adult animals. One pool of genomic DNA was used to construct a library for 454 sequencing and the other used for Illumina paired-end sequencing. The 454 sequencing resulted in 8.1 million reads (2.7 Gb), which were assembled into contigs using the Phusion assembler [60]. The Illumina run resulted in 2.8 million paired end reads, which combined with the 454 data, was used to generate 5,100 scaffolds (scaffold N50 of 187 kb), resulting in a total coverage of ~50×.

The *Mnemiopsis *genome was scanned *in silico *for genes of interest using a reciprocal BLAST approach. Human, frog, *Drosophila *and *Nematostella *orthologs were used as queries for TBLASTN searches. Candidate matches were then used in BLASTP searches of the human genome to find the closest hit. If the closest match was not the original ortholog or if the E-value was greater than 0.001, then it was coded as being absent from the genome. A gene model was created by scanning the genomic region using Genscan [61]. This predicted protein sequence was then searched for conserved Pfam domains using SMART [62]. For certain genes of interest, gene-specific primers were designed for RACE PCR (MacVector, Cary, NC, USA). RACE PCR fragments were then conceptually spliced and aligned back to genomic contigs for comparison of exon-intron boundaries, using Sequencher (Gene Codes, Ann Arbor, MI, USA).

### Phylogenetic analyses

The *Mnemiopsis *predicted amino acid sequences were aligned with the sequences of other organisms. The predicted domains or regions of interest were trimmed and aligned using Muscle, then corrected by hand for alignment errors (see Additional file 3, Additional file 4). Bayesian phylogenetic analyses were performed using MrBayes 3.1.2 [63] using the 'mixed' amino acid model with four independent runs of 5 million generations each, sampled every 100 generations with four chains. A summary consensus tree was produced in MrBayes from the last 49,000 trees of each run (196,000 trees in total), representing 4,900,000 stationary generations. Posterior probabilities were calculated from this consensus. Maximum likelihood analyses were performed using PhyML [64], using the WAG model with 1000 bootstraps. Alignments and nexus files are available upon request.

## Competing interests

The authors declare that they have no competing interests.

## Authors' contributions

KP designed the study, performed the genome search and phylogenetics, isolated genes, performed expression analyses, and drafted the manuscript. JFR performed the genome search and phylogenetics. NISC performed sequencing. JCM assembled the Mnemiopsis genome. ADB helped to design the study and helped in manuscript preparation. MQM participated in study design and manuscript preparation. All authors read and approved the final manuscript.

## Supplementary Material

Additional file 1 **Bayesian consensus tree with sponge sequences**. Bayesian consensus tree including sponge and *Trichoplax *Wnt sequences. *Mnemiopsis *genes are marked by arrows and shaded in red, and sponge and *Trichoplax *sequences are marked by asterisks. Taxa abbreviations are as follows: Aqu = *Amphimedon queenslandica*; Ate = *Archaearanea tepidarium*; Bfl = *Branchiostoma floridiae*; Cte = *Capitella teleta*; Hsa = *Homo sapiens*; Lgi = *Lottia gigantea*; Mle = *Mnemiopsis leidyi*; Nve = *Nematostella vectensis*; Pte = *Archaearanea tepidariorum*; Sko = *Saccoglossus kowalevskii*; Spu = *Strongylocentrotus purpuratus*; Tad = *Trichoplax adhaerens*; Tca = *Tribolium castaneum*. Click here for file

Additional file 2 **Genomic contigs of Wnt pathway components**. Text file containing genomic contigs of additional Wnt components that were identified *in silico*. Also listed are the Genscan predictions of protein sequences. Click here for file

Additional file 3 **Alignment used in Figure **4. Nexus/text file of alignment used for analyses in Figure 4. Click here for file

Additional file 4 **Alignment with additional taxa**. Nexus/text file of alignment used for analyses in Additional file 1. Click here for file
