# Supplementary figures and images for: Genomic insights into Wnt signaling in an early diverging metazoan, the ctenophore Mnemiopsis leidyi
Source: EvoDevo. 2010 Oct 4;1:10. doi: 10.1186/2041-9139-1-10 (PMC2959043; doi:10.1186/2041-9139-1-10)

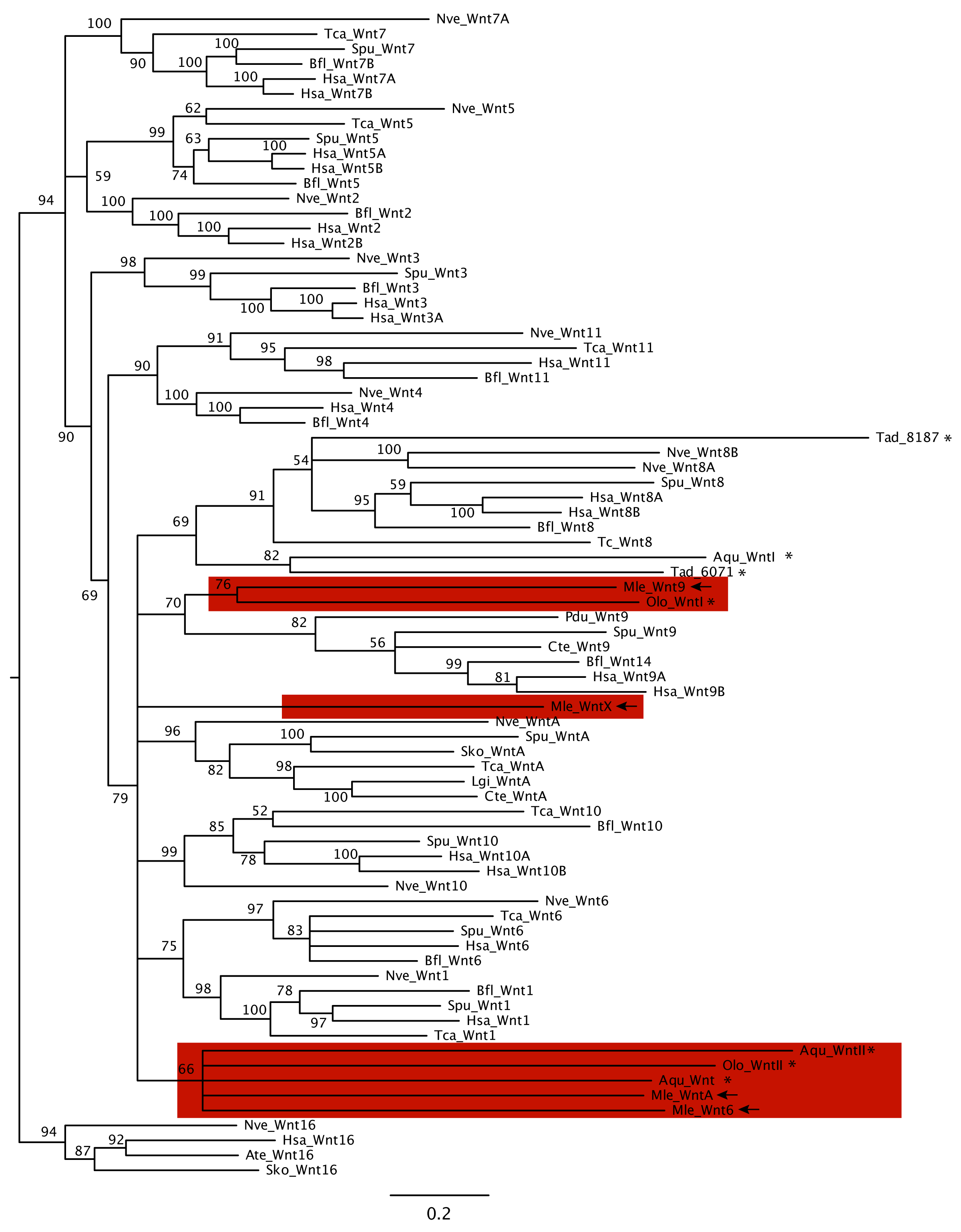

Supplement: Additional file 1 — Bayesian consensus tree with sponge sequences. Bayesian consensus tree including sponge and Trichoplax Wnt sequences. Mnemiopsis genes are marked by arrows and shaded in red, and sponge and Trichoplax sequences are marked by asterisks. Taxa abbreviations are as follows: Aqu = Amphimedon queenslandica; Ate = Archaearanea tepidarium; Bfl = Branchiostoma floridiae; Cte = Capitella teleta; Hsa = Homo sapiens; Lgi = Lottia gigantea; Mle = Mnemiopsis leidyi; Nve = Nematostella vectensis; Pte = Archaearanea tepidariorum; Sko = Saccoglossus kowalevskii; Spu = Strongylocentrotus purpuratus; Tad = Trichoplax adhaerens; Tca = Tribolium castaneum. [file 2041-9139-1-10-S1.TIFF]
